# Improved prediction of conopeptide superfamilies with ConoDictor 2.0

**DOI:** 10.1093/bioadv/vbab011

**Published:** 2021-06-17

**Authors:** Dominique Koua, Anicet Ebou, Sébastien Dutertre

**Affiliations:** 1 Bioinformatic Team, Département Agriculture et Ressources Animales, Institut National Polytechnique Félix Houphouët-Boigny, Yamoussoukro, BP 1093, Côte d’Ivoire; 2 Institut des Biomolécules Max Mousseron, Université de Montpellier, CNRS, ENSCM, Montpellier, 34095, France

## Abstract

**Motivation:**

Cone snails are among the richest sources of natural peptides with promising pharmacological and therapeutic applications. With the reduced costs of RNAseq, scientists now heavily rely on venom gland transcriptomes for the mining of novel bioactive conopeptides, but the bioinformatic analyses often hamper the discovery process.

**Results:**

Here, we present ConoDictor 2.0 as a standalone and user-friendly command-line program. We have updated the program originally published as a web server 10 years ago using novel and updated tools and algorithms and improved our classification models with new and higher quality sequences. ConoDictor 2.0 is now more accurate, faster, multiplatform and able to deal with a whole cone snail venom gland transcriptome (raw reads or contigs) in a very short time. The new version of Conodictor also improves the identification and subsequent classification for entirely novel or relatively distant conopeptides. We conducted various tests on known conopeptides from public databases and on the published venom duct transcriptome of *Conus geographus*, and compared previous results with the output of ConoDictor 2.0, ConoSorter and BLAST. Overall, ConoDictor 2.0 is 4 to 8 times faster for the analysis of a whole transcriptome on a single core computer and performed better at predicting gene superfamily.

**Availability and implementation:**

ConoDictor 2.0 is available as a python 3 git folder at https://github.com/koualab/conodictor.

**Supplementary information:**

Supplementary data are available at *Bioinformatics Advances* online.

## 1 Introduction

Cone snails are among the richest sources of biologically active compounds (Olivera *et al.*, 2014). The major component of their venom is made of disulfide-rich peptides with the neurotoxic activity known as conotoxins. They are prey-type specific and target a wide range of ion channels and other membrane proteins, providing pharmacological, physiological and therapeutic compounds of interest (Gao *et al.*, 2017; Prashanth *et al.*, 2014). For an in-depth study of cone snail venom, researchers have in recent years implemented transcriptomics, proteomics, biochemistry, physiology and bioinformatics into an accelerated discovery strategy known as venomics (Dutertre, 2014). Venomics involves the prediction and classification of putative conotoxins at the transcriptome level. This step traditionally relies on a time-consuming homology-based BLAST (Camacho *et al.*, 2009) search against a specialized database, which often returns incomplete results and false positives. To overcome this limitation, several tools have been created. First, ConoPrec is a web-based tool that assigns precursors into conopeptides superfamilies using sequence similarity of the signal sequence. Although it was used to report on the discovery of new conotoxins in the venom of *Califorconus californicus* (Kaas *et al.*, 2012), it cannot analyze large batches of datasets such as whole transcriptomes. ConoSorter is a discovery pipeline based on regular expression matching and the use of hidden Markov models to identify and classify conotoxins precursors (Lavergne *et al.*, 2013). This program still requires significant manual inputs from the user in order to sort out the results, especially for novel or divergent sequences. Finally, the ConusPipe is based on the combination of machine learning models with cross-species BLAST to retrieve a list of putative conotoxins (Fu *et al.*, 2018).

Here we present ConoDictor 2.0, an update of the webserver ConoDictor (Koua *et al.*, 2012). It is based on the complementary predictions of an updated set of generalized models (Position-Specific Scoring Matrix, PSSM) and profiles hidden Markov models (pHMMs) to predict conotoxins and their classification into conotoxins gene superfamily (Koua *et al.*, 2013).

## 2 Methods

### 2.1 Algorithm improvements

Conopeptide precursors are generally made of three parts: a signal peptide, a propeptide region and a mature sequence. The gene superfamily assignment is based on the highly conserved signal peptide. However, the cysteine framework of the mature peptides associated with a given superfamily is also remarkably stable. As for the previous version of ConoDictor, the *insilico* classification algorithm is based on a combination of predictions made by profiles (PSSMs and pHMMs) designed from all three parts of the precursor sequence. However, ConoDictor 2.0 algorithm implements an optional filtering step where sequences are discarded if they do not match at least a mature peptide profile either for PSSMS or HMMs. This step allows removing spurious matches of pHMMs and PSSMs that tend to report useless fragments of signal or propeptides as well as false positives.

### 2.2 Data acquisition and preprocessing

Conopeptides sequences data were downloaded from UniProt database version 2019_10 (The UniProt Consortium, 2019). We excluded the data from conoserver because of some errors in the annotation of sequences and to create a reliable training set different from ConoPrec’s classifier training set. Only full precursors with superfamily classification annotation were retained. Nonexperimentally verified sequences with ‘POTENTIAL’, ‘HYPOTHETICAL’ or ‘SYNTHETIC’ keywords were discarded. This produced a final dataset of 727 sequences (Supplementary File S1). The gene superfamilies classification of each sequence was extracted from UniProt sequence annotation. Superfamilies with at least 3 members (19 superfamilies) were considered, while superfamilies with fewer members (E, F, Q, V, Y) were removed from the dataset. Each sequence was then divided into functional parts using UniProt’s annotation: signal, propeptide, mature peptide. Furthermore, sequences were divided for consistent alignments (less gaps, better cysteine framework). This approach improves the scores and reduces conflicts. Sequences from each of the 19 first gene superfamilies resulted in 79 files to be aligned (Supplementary File S2). For each gene superfamily, the final dataset was randomly split into two parts: the training set composed of 75% of sequences and the test set containing 25%. Sequences were aligned with MAFFT v7.471 (Katoh and Standley, 2013). The multiple sequence alignments obtained were further used for model construction, very minor manual refinements of these alignments were necessary.

### 2.3 Models construction

Using the training set, profile hidden Markov models were constructed for each protein region of a superfamily using the hmmbuild tool from HMMER v3.3 (Eddy, 2011) with default parameters. The resulting pHMMs were further validated against the test sets using hmmsearch with an *e*-value significance of 0.1.

For generalized profiles, sequences were first weighted using the Voronoi weighting strategy of the pfw tool (from pftool v3.2). Generalized profiles were constructed using the pfmake tool (from pftool v3.2) in semi-global mode (Schuepbach *et al.*, 2013). Obtained profiles were then calibrated against Uniprot randomized sequences with pfcalibrateV3 and cut-off values tuned manually.

## 3 Results

ConoDictor 2.0 is implemented in Python3 and distributed under the GPL-3 license. The previous version was a web Perl-CGI application (no longer supported). The new version is a portable standalone application that one can easily run locally on a ‘standard’ computer regardless of the operating system. ConoDictor 2.0 is also available on BioContainers (https://github.com/BioContainers/containers.git), as a docker image (https://hub.docker.com/r/ebedthan/conodictor) and also as a singularity image for use with supercomputers or clusters. The only input ConoDictor 2.0 requires is the assembled transcriptome or the raw reads file either in DNA or amino acid: used alphabet is automatically recognized. ConoDictor 2.0 run predictions directly on the proteins file (submitted or dynamically generated) and tries to report the longest conopeptide precursor-like sequence. Detailed settings and command-line options can be found on the ConoDictor homepage (https://github.com/koualab/conodictor.git).

### 3.1 Updated HMM and PSSM profiles for better superfamily prediction

We analyzed the current gene superfamily members and propose an extensive set of HMM and PSSM profiles that better describe them. For example, the superfamily M is now characterized by eight profiles: one for the signal peptide, two for propeptides and five for the mature peptide. Implementing such a strategy resulted in an improved score and in the reduction of classification conflicts. ConoDictor 2.0 is therefore shipped with 158 profiles (HMM and PSSM) instead to the 48 profiles in Conodictor version 1. We also report in Supplementary File S3 a list of misclassified conopeptides and suggestions on some conopeptides superfamilies.

### 3.2 Predicting a sequence to be a conopeptide

As a first test, we evaluated ConoDictor 2.0 against a custom dataset (Supplementary File S4) composed of a set of known and correctly annotated conotoxins (representing our true positive dataset) and some random sequences of venomous animals (spiders, snakes, sea urchins, jellyfish) available on UniProtKB (to represent the true negative dataset). ConoDictor 2.0 was evaluated along with ConoSorter v1.1 and BLAST v2.9.0+. We excluded ConoPrec as it was not available as a standalone version and ConusPipe as we did not find enough information to have it properly installed. In this evaluation, ConoDictor 2.0 demonstrated the highest power to distinguish conopeptides and achieve the overall highest specificity (Table 1).

**Table 1.** Evaluation on predicting a sequence as a conopeptide

Tool	Sensitivity (%)^a^	Specificity (%)^b^
ConoDictor 2.0	98.31	96.72
BLAST 2.9.0+	100	4.91
ConoSorter 1.1	97.29	1.19

a Sensitivity refers to the proportion of conotoxins from the true-positive dataset classified as conotoxin by the given tool. Sensitivity = number of classified as conotoxin * 100/size of the true-positive dataset.

b Specificity refers to the proportion of sequences from the true-negative dataset classified as nonconotoxin by the given tool. Specificity = number of classified as nonconotoxin * 100/size of the true-negative dataset.

### 3.3 Classifying a conopeptide in its gene superfamily

We evaluated ConoDictor 2.0 on another custom dataset (Supplementary File S5) composed of 290 conopeptides from known gene superfamilies. These 290 sequences were not used in the training phase. To test these 290 conopeptides, we added 250 unreviewed cone snails housekeeping genes from TrEMBL which logically do not have gene superfamily annotation. We used the same pool of tools for comparison. ConoDictor 2.0 correctly assigned 287 out of 290 conopeptides into their correct known gene superfamily. This classification was confirmed by manual validation. The sensitivity and specificity of this testing phase are reported in Table 2.

**Table 2.** Evaluation on the classification of conopeptides in a superfamily

Tool	Sensitivity (%)	Specificity (%)
ConoDictor 2.0	98.96	100
BLAST 2.9.0+	100	30.4
ConoSorter 1.1	96.63	46.4

### 3.4 Re-analysis of *Conus geographus* transcriptome

**Fig. 1.**
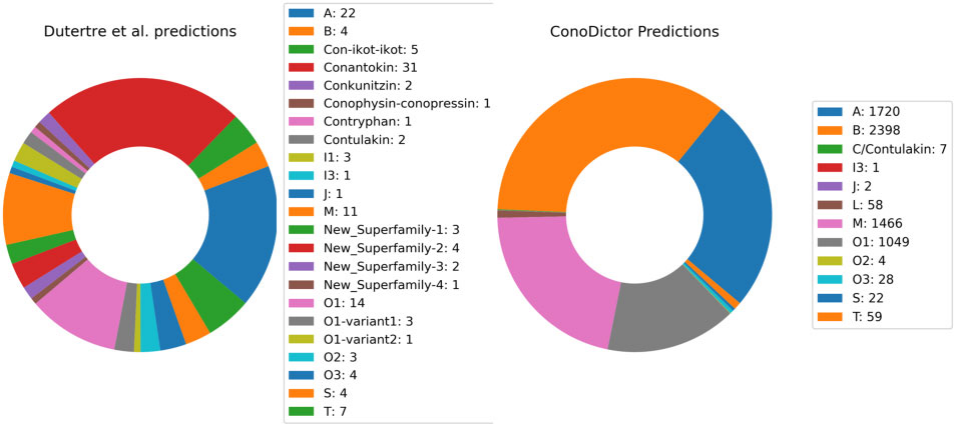
Distribution of conopeptides superfamilies identified by Dutertre *et al.*, versus ConoDictor 2.0

To further evaluate ConoDictor 2.0, we used the published *C*.*geographus* venom gland transcriptome (Dutertre *et al.*, 2014). The comparison was made with BLAST and ConoSorter using one computer core. We ran ConoDictor 2.0 using custom parameters to fit conditions used by Dutertre *et al.*: we kept only sequences starting with methionine and ending with a stop codon, with a minimum read length of 50 bp, considered a minimum number of read occurrences of 2. ConoDictor 2.0 ran in 2 min 31 s. Results obtained in both studies are presented in Figure 1.

We furthermore predict gene superfamilies of conopeptides reported by Dutertre *et al.* using ConoDictor 2.0. Results show that ConoDictor performed very well on the classification task (Table 3).

**Table 3.** Comparison of conopeptides classification by both studies

Superfamily	Classified by Dutertre *et al.*	Classified by ConoDictor 2.0
A	22	22
B	3	0
Conantokins	31	31
Con-ikot-ikot	5	0
Conkunitzin	2	0
Conophysin-Conopressin	1	0
Contryphan	1	0
C/Contulakin	2	2
I1	3	3
I3	1	1
J	1	1
M	11	11
O1	18	18
O2	3	3
O3	4	4
S	4	3
T	7	7
New superfamily 1	3	0
New superfamily 2	4	0

### 3.5 Improved outputs

**Fig. 2.**
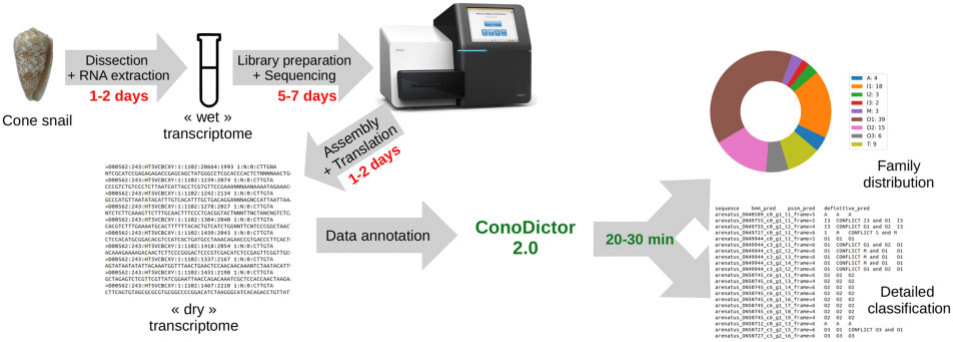
ConoDictor 2.0 shortens conopeptide discovery time and improves visualization of gene superfamily distribution in the analyzed transcriptome

In addition to improving analysis speed and quality, ConoDictor 2.0 provides the user outputs helping to rapidly validate newly discovered conopeptides. Conodictor 2.0 outputs a classification chart of peptides distribution in the submitted dataset (possibly the venom gland transcriptome) and allows FASTA format exportation of matched sequence regions (Fig. 2).

## 4 Discussions

Compared to the initial web-based version, the new standalone Conodictor 2.0 ensures, among others, the two following advantages. (i) One can run offline analyses implying big input files without bandwidth transfer limitations: when we consider a full transcriptome could be up to 2 or 4 GB, sending such file to the server may result in additional cost or issues for users. (ii) Results are kept client-side: running a home installed application fully ensures data privacy and long-term availability. Indeed, data storage quickly becomes a bottleneck when providing a web service. A standalone version, therefore, guarantees free usage of the proposed application.

Compared to the commonly used BLAST v2.9.0+ and ConoSorter 1.1, the new ConoDictor 2.0 allows to properly discriminate between conotoxin and nonconotoxin. On our test set, nearly 97% of nonconotoxin sequences were recognized as such by ConoDictor 2.0 when BLAST and ConoSorter wrongly considered more than 95% of true negatives as conotoxins. Moreover, Conodictor 2.0 is nearly 99% efficient when it is to associate an identified conotoxin in its correct gene superfamily. Building several specific models for a single family greatly improved specificity.

ConoDictor 2.0 is based on updated pHMMs and PSSMs that have been trained with better quality sequences deposited since the previous version, 10 years ago. Moreover, the tool is now using updated tools (e.g. pftools 3.2), which are faster and able to deal with larger files. Conodictor 2.0 can also be run on multiple cores, thus giving the capability to accelerate the prediction of conopeptides superfamilies in a whole transcriptome file.

Compared to what can be done manually, for the analysis of *C.geographus* venom gland transcriptome, ConoDictor 2.0 was able, in <3 min, to predict and classify the majority of the sequences identified by Dutertre *et al.* Moreover, Conodictor 2.0 reported more sequences. However, ConoDictor 2.0 misclassified the three conopeptides identified as belonging to I1 superfamily by Dutertre *et al.* This can be explained by the fact that, even if the signal sequence of these conopeptides is similar to one of known and reviewed I1, there is a missing C in the signal peptide of these three conopeptides. In addition, the three reported conopeptides have a different cysteine framework compared to the one of the publicly available I1 conopeptides. Furthermore, Dutertre *et al*. also reported 3 B conopeptides that further investigations lead us to the conclusion that they were not from the B superfamily since reported B have a very different signal peptides from the publicly available B. Finally, ConoDictor 2.0 could not report the classification of conopeptides identified as ‘new superfamily’ (that should be decided a proper name), Conomarphin, or Conophysin by Dutertre *et al*. because there is still no model to predict these families. Nevertheless, in the final ConoDictor 2.0 models, we added the HMMs and PSSMs models of Con-ikots-ikots, Conkunitzin and Conophysins families to enable automated prediction of these families.

Concerning the prediction output, ConoDictor 2.0 can report either all matched fragments (enabled by default) or only ‘full precursor-like matches’ composed of signal peptide, propeptide and mature peptide. This allows a fast and easy manual check from the final user.

ConoDictor 2.0 outputs a summary text (tab-separated value) file of sequence identifiers with the predicted superfamily. We also provide a distribution of conopeptides gene superfamilies as a donut graph. Moreover, the user can activate an option to export a FASTA format list of identified and classified sequences.

## 5 Conclusion

In this study, we presented the new ConoDictor 2.0, proposed as a standalone python3 command-line tool that has been improved by an updated set of pHMMs and PSSMs. Conodictor 2.0 is now able to efficiently predict conotoxins from a whole assembled *Conus* venom gland transcriptome in less than 10 min.

## Funding

None declared.

*Conflict of Interest*: none declared.

## Supplementary Material

vbab011_Supplementary_Data
