# Supplementary material for: Improved prediction of conopeptide superfamilies with ConoDictor 2.0
Source: Bioinform Adv. 2021 Jun 17;1(1):vbab011. doi: 10.1093/bioadv/vbab011 (PMC9710579; doi:10.1093/bioadv/vbab011)
Supplement: vbab011_Supplementary_Data [file vbab011_supplementary_data.zip › Supplementary Materials for Online/supplementary_file3.docx]

**Supplementary file 3**

Reported sequences in this file are misclassified or have some of their precursors regions too different from others superfamily members. We therefore propose to remove them from their current superfamily.

| Superfamily | Accession numbers |
| --- | --- |
| A | C6ZJQ2, D4HPD6, O77256 |
| B | J7JU64 |
| H | W4VSG7 |
| M | B2KPN7, B3SVF0, B3SVF1 |
| T | P69765, Q9BP46 |
